# MEMS Oscillators‐Network‐Based Ising Machine with Grouping Method

**DOI:** 10.1002/advs.202310096

**Published:** 2024-05-02

**Authors:** Yi Deng, Yi Zhang, Xinyuan Zhang, Yang Jiang, Xi Chen, Yansong Yang, Xin Tong, Yao Cai, Wenjuan Liu, Chengliang Sun, Dashan Shang, Qing Wang, Hongyu Yu, Zhongrui Wang

**Affiliations:** ^1^ Department of Electrical and Electronic Engineering The University of Hong Kong Pokfulam Road Hong Kong 999077 China; ^2^ ACCESS ‐ AI Chip Center for Emerging Smart Systems InnoHK Centers Hong Kong Science Park Hong Kong 999077 China; ^3^ School of Microelectronics Southern University of Science and Technology Shenzhen 518055 China; ^4^ Department of Electronic and Computer Engineering Hong Kong University of Science and Technology Hong Kong 999077 China; ^5^ Institute of Technological Sciences Wuhan University Wuhan 430072 China; ^6^ Institute of Microelectronics Chinese Academy of Sciences Beijing 100029 China

**Keywords:** combinatorial optimization, Ising machine, Max‐Cut, MEMS oscillator, semidefinite programming relaxation

## Abstract

Combinatorial optimization (CO) has a broad range of applications in various fields, including operations research, computer science, and artificial intelligence. However, many of these problems are classified as nondeterministic polynomial‐time (NP)‐complete or NP‐hard problems, which are known for their computational complexity and cannot be solved in polynomial time on traditional digital computers. To address this challenge, continuous‐time Ising machine solvers have been developed, utilizing different physical principles to map CO problems to ground state finding. However, most Ising machine prototypes operate at speeds comparable to digital hardware and rely on binarizing node states, resulting in increased system complexity and further limiting operating speed. To tackle these issues, a novel device‐algorithm co‐design method is proposed for fast sub‐optimal solution finding with low hardware complexity. On the device side, a piezoelectric lithium niobate (LiNbO_3_) microelectromechanical system (MEMS) oscillator network‐based Ising machine without second‐harmonic injection locking (SHIL) is devised to solve Max‐cut and graph coloring problems. The LiNbO_3_ oscillator operates at speeds greater than 9 GHz, making it one of the fastest oscillatory Ising machines. System‐wise, an innovative grouping method is used that achieves a performance guarantee of 0.878 for Max‐cut and 0.658 for graph coloring problems, which is comparable to Ising machines that utilize binarization.

## Graphical abstract

A novel grouping method, which eliminates the need of second‐harmonic injection locking (SHIL), is proposed for micro‐electromechanical system (MEMS) oscillator network‐based Ising machine. This grouping method achieves a performance guarantee of 0.878 for Max‐cut and 0.658 for graph coloring problems. Besides, the MEMS oscillator operates at speeds greater than 9 GHz, making it one of the fastest oscillatory Ising machines.

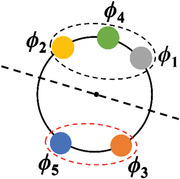

## 1 Introduction

Combinatorial optimization (CO) problems play a vital role in various fields such as operations research, computer science, and artificial intelligence. Many of these CO issues fall into the category of nondeterministic polynomial‐time (NP)‐complete or NP‐hard problems, which can only be verified, not solved, within polynomial time on a digital computer. Furthermore, as the scale of these problems increases, the energy and time required to solve them on computers with von Neumann architecture grow exponentially. This challenge is further compounded by the slowing down of Moore's law, which has driven the progress of digital electronics for several decades.

To address these challenges, numerous novel solvers have been developed,^[^ ^1^, ^2^, ^3^, ^4^, ^5^, ^6^, ^7^, ^8^, ^9^, ^10^ ^]^ including physical Ising machines.^[^ ^11^, ^12^, ^13^ ^]^ In contrast to conventional digital computers, physical Ising machines draw inspiration from computational methods in statistical physics and thermodynamics. They can solve Ising problems by employing their continuous‐time internal physical evolution process, which minimizes the system energy and equivalently finds the solution to the CO problems.

Various physical Ising machines have been prototyped in recent years, such as the D‐WAVE quantum Ising machine,^[^ ^14^, ^15^ ^]^ coherent Ising machine,^[^ ^16^, ^17^, ^18^, ^19^ ^]^ and oscillator‐network‐based Ising machine.^[^ ^20^, ^21^, ^22^ ^]^ Among them, the oscillator‐network‐based Ising machine offers several benefits, including a compact footprint and low power dissipation. Devices like VO_2_ threshold switches^[^ ^23^, ^24^ ^]^ and CMOS^[^ ^25^, ^26^, ^27^ ^]^ are used to construct the oscillators. However, oscillator‐based Ising machines face two challenges: a relatively low oscillating frequency and the need for an external second harmonic injection locking (SHIL) signal^[^ ^20^, ^21^, ^22^, ^23^, ^24^, ^28^, ^29^, ^30^, ^31^, ^32^, ^33^, ^34^ ^]^ to ensure the oscillator system aligns with the Ising model.^[^ ^35^ ^]^ This binarization can result in increased system complexity and limited operating frequency.

To increase the oscillating frequency, we utilize LiNbO_3_, a common piezoelectric material, to build the MEMS oscillator network due to its high working frequency and stability.^[^ ^36^, ^37^, ^38^, ^39^ ^]^ This leads to a shorter time‐to‐solution. Kourani et al. fabricated a MEMS resonator using a suspended Z‐cut LiNbO_3_ thin film, achieving 38.7 GHz using the ninth‐order asymmetric (A9) Lamb‐wave modes. They also demonstrated a 12.9 GHz Pierce oscillator utilizing the third overtone (A3) mode,^[^ ^38^ ^]^ aligning with the upper echelons of reported operational frequencies. Recent advances in MEMS oscillator innovation, notably the utilization of resonant‐fin‐transistor (RFT) architectures, have yielded operational frequencies surmounting the 30 GHz threshold,^[^ ^40^, ^41^ ^]^ which reveals the potential to significantly speed up Ising machines. Additionally, to mitigate the SHIL requirement, we introduce a novel grouping method for the oscillator‐network‐based Ising machines. Our method can achieve high‐quality sub‐optimal solutions with low system complexity while maintaining a high frequency. For instance, our approach can obtain a performance guarantee of 0.878 for Max‐cut and 0.658 for graph coloring problems.

## 2 Results and Discussion

### 2.1 LiNbO_3_ A3 Mode MEMS Resonators and Pierce Oscillators

**Figure 1:**
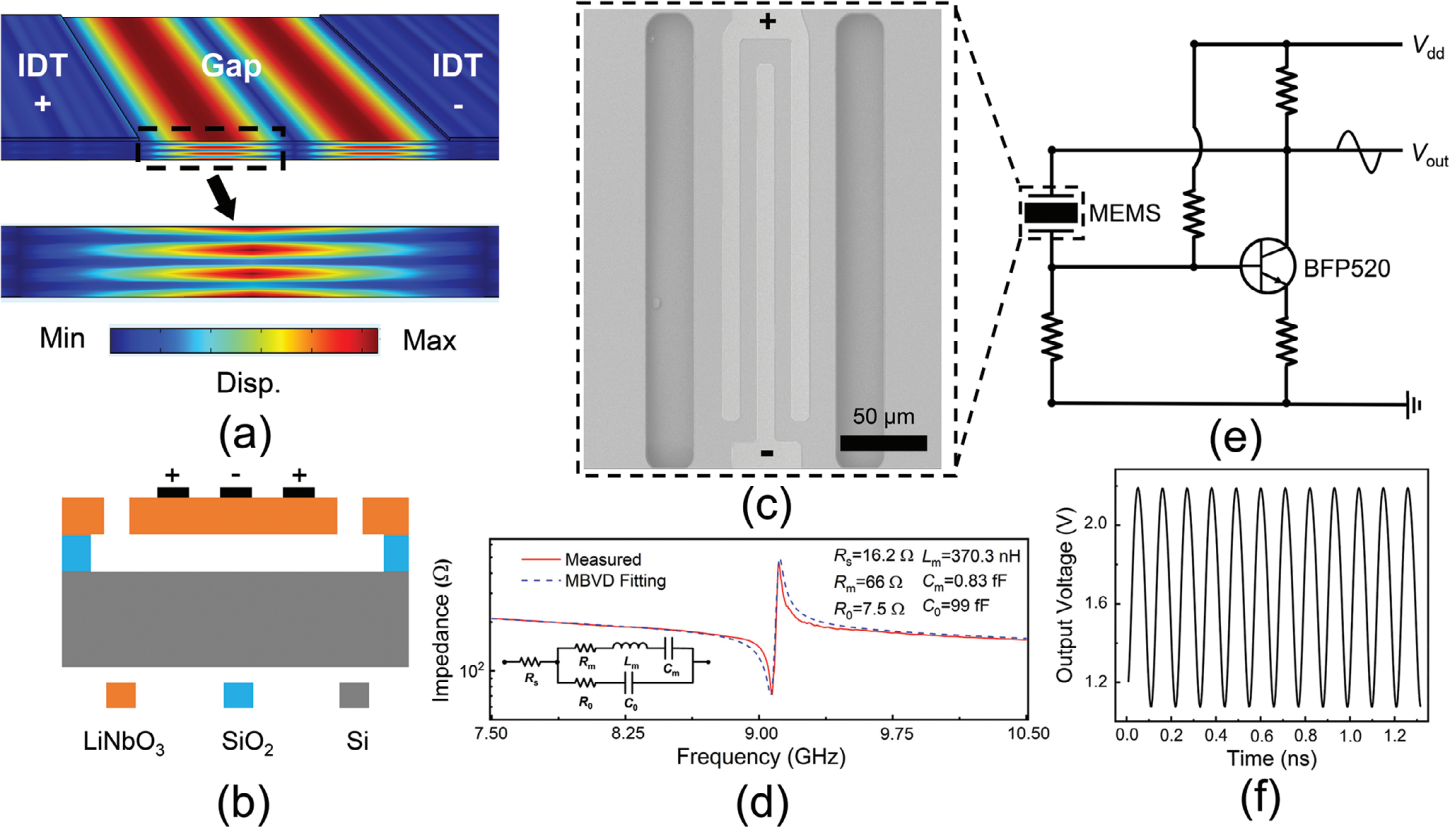
a) Simulated displacement field of A3 Lamb mode. b) Schematic diagram and c) SEM of the fabricated A3 mode LiNbO_3_ resonator. d) The measured and the MBVD model fitted impedance curves, the inset shows the equivalent MBVD circuit diagram of the LiNbO_3_ resonator. e) The schematic of the MEMS Pierce oscillator circuitry. f) The output waveform of the designed Pierce oscillator.

The A3 Lamb mode is a type of antisymmetric Lamb mode, characterized by a displacement that is antisymmetric with respect to the median plane of the wave. This results in equal magnitudes of vertical displacement but in opposite directions for the longitudinal components on either side of the median plane, as demonstrated in **Figure** **1**a through 3D eigen‐frequency finite‐element analysis (FEA). In the present study, A3 mode MEMS resonators were constructed utilizing a 600 nm ZX‐LiNbO_3_ thin film. Three interdigital electrodes (IDTs) were employed for the excitation of acoustic waves, with both the width and spacing of the IDTs set as 10 µm. Figure 1b,c displays the schematic diagram and scanning electron micrograph (SEM) of the fabricated device, respectively. Then the device was characterized using a Keysight network analyzer, with both measured and modified Butterworth‐Van Dyke (MBVD)‐fitted impedance curves displayed in Figure 1d. The device features a resonant frequency of 9.1 GHz, enabling high‐frequency oscillation. It also exhibits *Q* factors of approximately 300 (*Q*_s_ = 288, *Q*_p_ = 355). The extracted parameters—*R*_s_, *R*_m_, *R*_0_, *C*_m_, *C*_0_, and *L*_m_—were then used to design Pierce oscillators using the Pspice circuit simulator. Pierce oscillators are widely employed in crystal and MEMS resonator circuits due to their stable frequency and straightforward design. As depicted in Figure 1e, the Pierce oscillator has a reduced set of external components and a grounded source configuration. The MEMS resonator serves as a crucial frequency control component for generating stable frequency outputs. In this study, an Infineon BFP520 transistor with a 45 GHz cutoff frequency (*f*_t_) was utilized as the amplifier to sustain continuous oscillation, and the supply voltage (*V*_dd_) was set to 2.5 V. The oscillator's output, with a period (*T*) of approximately 0.11 ns, is presented in Figure 1f. Each individual oscillator consumes 30 mW of power. It is worth noting that this paper does not take the influence of parasitic effects due to packaging and oscillator integration into account. Such parasitic and self‐resonance become pronounced at high frequency. Actually, within the LiNbO_3_ antisymmetric Lamb wave resonator, aside from the A3 mode, additional modes such as A1 and A5 coexist. The self‐resonance stemming from parasitic effects can be likened to an additional mode. To minimize undesired consequences, an amplifier with a suitable bandpass transfer function can be employed. This approach allows for the excitation of the A3 mode while simultaneously suppressing A1 and other higher‐order modes, as well as the parasitic part. Achieving optimal performance in an oscillator necessitates a careful balance between optimizing the oscillator's performance, effectively managing power consumption, and refining the overall circuit design. Further detailed information on this subject can be found in ref. [42]. Besides, phase noise matters in practical implementation. An optimal level of noise is essential for Ising machine to introduce nonzero temperature perturbation to the system to escape local optima and converge towards the global optimal solution.

### 2.2 Ising Model and Grouping Method

**Figure 2:**
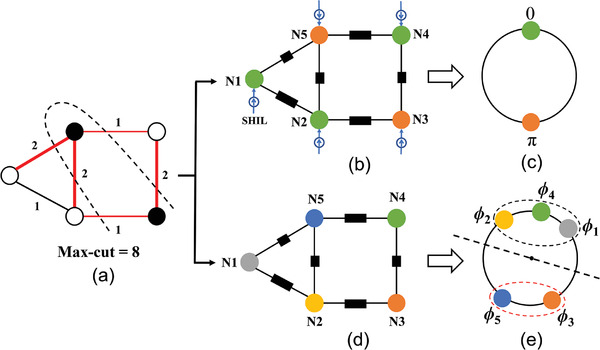
a) Illustration of the Max‐cut problem. b) Mapping the Max‐cut problem to an oscillators‐network‐based Ising machine with SHIL. c) Solution to the problem with binarized phases, 0 and π. d) Mapping the Max‐cut problem to an oscillators‐network‐based Ising machine without SHIL. e) Solution to the problem with grouping method.

The Max‐Cut problem poses the question: Given a weighted, undirected graph with vertices and edges, how can the vertices be divided into two groups such that the total weight of the edges crossing between the groups is maximized?^[^ ^43^ ^]^ The weight of the edges connecting vertices in different groups is referred to as a “cut,” and the maximized total weight is known as the maximum cut (**Figure** **2**a). It has been demonstrated that the Max‐Cut problem, an NP problem, can be directly mapped to equivalent Ising Hamiltonian equations^[^ ^44^ ^]^ (see Supporting Information).

The Ising model, proposed by Wilhelm Lenz and Ernst Ising in the 1920s as a mathematical model for describing ferromagnetism in substances,^[^ ^45^ ^]^ consists of a set of discrete variables representing the magnetic moment or spins, which take on values of +1 or ‐1 corresponding to spin up or down.^[^ ^46^ ^]^ The interaction energies between each pair of spins are calculated to obtain a simplified Ising Hamiltonian energy function for the entire system, which can be expressed as follows:

(1) $$H\;=\;-\sum_{i\leq j\leq n}^{n}J_{ij}s_{i}s_{j}$$

where *H* denotes the Hamiltonian operator, *s_i_* and *s_j_* represent the states of spin *i* and *j*, *n* is the total number of spins, and *J_ij_* is the coupling coefficient. This equation is often used to solve CO problems by assigning appropriate coupling coefficients to address specific issues.^[^ ^47^ ^]^ The optimal solution corresponds to the spin configuration with the lowest system energy *H*_min_, which can be determined from the spin states.

In the oscillator‐network‐based Ising machine, each oscillator acts as a spin node, and the coupling between spin nodes is achieved through various mediums, such as resistances or active components (Figure 2b). In an oscillator network where all oscillators are identical and generate cosine signals, the entire system energy is governed by the Lyapunov function.^[^ ^48^ ^]^ Consequently, the global energy function can be described as follows:

(2) $$\begin{array}{cc} E\;\left(\vec{\Delta \phi }\left(t\right)\right) & =-\sum_{i,\;j,j\neq i}^{n}J_{ij}\cos\left(\phi_{i}\left(t\right)-\phi_{j}\left(t\right)\right)\\ & =\;-\sum_{i,\;j,j\neq i}^{n}J_{ij}\cos\left(\Delta \phi_{ij}\left(t\right)\right) \end{array}$$

where $\vec{\Delta \phi }\left(t\right)$ represents different phase configurations, *J_ij_* is proportional to the strength of coupling between oscillators *i* and *j*, *n* is the number of oscillators, and Δ*ϕ_ij_* (*t*) is the phase difference between oscillators *i* and *j*. Since the system energy is determined by the global Lyapunov function, the derivative of energy with respect to time is less than zero,^[^ ^46^ ^]^ causing the system energy to minimize automatically as it evolves over time. Comparing Equations (1) and (2), if Δ*ϕ_ij_* equals to 0 or ±π and then cos (Δϕ_*ij*_(*t*)) =  1 or cos (Δϕ_*ij*_(*t*)) =   − 1, the system energy function will accurately map the Ising Hamiltonian equation. However, oscillator phases are continuous values ranging from 0 to 2π, so the SHIL was introduced to constrain the phases of oscillators to two discrete values,^[^ ^21^ ^]^ such as 0 and π (Figure 2c). These two different phases map to binary values of *s_i_*, allowing the solution to be determined from the phases of oscillators.

The inclusion of SHIL signals leads to increased system complexity and additional power consumption. In the absence of SHIL signals (Figure 2d), oscillator phases are continuous and can be represented on a unit circle, as illustrated in Figure 2e. These phases can be randomly divided into two groups using a straight line passing through the center of the circle, with the effective angle range of this line spanning from 0° to 180°. The probability of two oscillators being in different groups is $\frac{\Delta \phi_{ij}}{\pi }$, while the probability of two oscillators being in the same group is $1-\frac{\Delta \phi_{ij}}{\pi }$. The value of *s_i_s_j_* is −1 and +1 when the two oscillators are in different groups and in the same group, respectively. Furthermore, for an *n*‐scale problem, this grouping method can produce a maximum of *n* solutions, each solution is associated with a specific angle range, which can be translated to its corresponding probability (see Supporting Information). The SHIL‐free Ising machine, using a grouping method, can achieve a minimum expectation value that is larger or equal to 87.8% of the true Max‐cut when solving positive‐weight Max‐cut problems (see Supporting Information), which means that there must be one solution being at least 87.8% of the optimal value and the solver has a performance guarantee of 0.878 in polynomial time. Notably, the derivation process closely resembles a high‐quality approximation algorithm that employs semidefinite programming (SDP) relaxation and randomized rounding,^[^ ^49^ ^]^ which are commonly used for combinatorial problems. In an improved algorithm presented in,^[^ ^50^ ^]^ the authors sought to find a phase configuration that minimizes energy, rather than solving a semidefinite program. The energy expression in their approach is similar to the energy function (2). Consequently, the SHIL‐free Ising machine with a grouping method can be considered a comparable approximation solver, where SHIL removal serves as a relaxation and the grouping method functions as randomized rounding.

### 2.3 Ising Machine's Performance

Firstly, the performance of an oscillator‐network‐based Ising machine without SHIL in solving Max‐cut problems is investigated. MEMS oscillators are employed as spin nodes to create oscillator networks, which map the corresponding graphs of Max‐cut problems. In order to simulate the convergence of coupled oscillators over a long period of time, we employ negative resistors in PSpice to model the negative coupling, which is functionally equivalent to the combination of ordinary resistors and inverters but simplifies solving process. And the coupling strength is related to the conductivity of the resistors. The resistors utilized for coupling are standardized at ‐1 kΩ, representing one unit weight. Additionally, cross coupling can be employed for negative coupling, albeit its applicability is primarily limited to ring oscillators.^[^ ^34^, ^51^ ^]^

**Figure 3:**
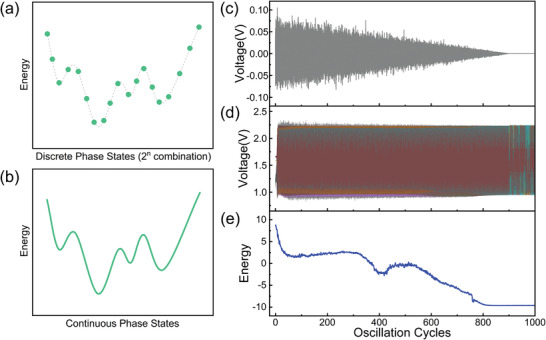
Schematic diagram of oscillator network energy landscape a) with SHIL and b) without SHIL. c) Illustration of the decaying noise. d) The waveforms of oscillators after adding the decaying noise. e) The corresponding system energy evolution with simulated annealing.

The simulated annealing method, which is essential for jumping out of the local energy minima, is extensively employed in traditional Ising machines. This approach enhances the probability of reaching the global minimum energy, thereby bolstering the overall performance of these systems. Apart from a constant term, Ising machines with and without the SHIL share similar global energy functions.^[^ ^21^ ^]^ However, the phase configurations for systems incorporating SHIL are discrete (**Figure** **3**a), while those without SHIL exhibit continuous configurations (Figure 3b). Local energy minima also exist in Ising machines without the SHIL component. Consequently, the solver proposed here also necessitates the utilization of the simulated annealing technique. A common approach for implementing simulated annealing in oscillator networks involves the injection of time‐varying noise, which diminishes over time. As illustrated in Figure 3c, a decaying noise signal is applied to an eight‐oscillator network. The temporal evolution of the oscillators' voltage is depicted in Figure 3d, from which the instantaneous phases can be extracted. Employing this phase data, the system energy of the Ising machine in accordance with Equation (2) is then calculated, revealing the energy evolution as presented in Figure 3e. The global energy is minimized following 800 oscillation cycles, signifying a solving time of 90 ns. The oscillators’ states eventually stabilize after the evolution, signifying the completion of the solving process. Unlike conventional Ising machines whose binary results can be directly read out, in this case, the solutions will be obtained after a grouping step, so the real solving time should consider the time for post‐data processing. However, it is noteworthy that the grouping is polynomial‐time‐consumed as the maximum number of solutions for an *n*‐scale Max‐cut problem is *n*, which will not significantly affect the solving speed. From all the possible solutions, the best one will be provided as the specific Max‐cut configuration which outputs at least 0.878 times of the maximum cutting. When problems scale up, the grouping step can be achieved by introducing a simple tool whose overhead is low and computational resources and time are acceptable.

**Figure 4:**
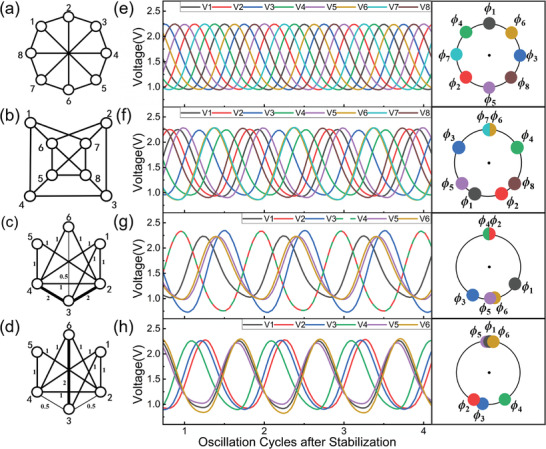
Illustration of a) the 8‐vertex graph with 12 edges, b) the 8‐vertex graph with 14 edges, c) the weighted 6‐vertex graph with 12 edges and d) another weighted 6‐vertex graph with 12 edges. e,f) The corresponding magnified oscillator waveforms and phase configuration of (a–d).

**Figure** **4**a displays an 8‐vertex graph with 12 edges, and the magnified graph in Figure 4e (left) exhibits the final states of the oscillators, from which we can derive a phase configuration consisting of continuous phases rather than two discrete phases on a unit circle (Figure 4e, right). After applying the grouping method as demonstrated in Figure 2e, several groups of solutions are produced. **Table** **1** lists the possible solutions and the corresponding angle ranges, probabilities, and the number of cuts. Due to the rotationally symmetric phase configuration in this case, all solutions obtained through grouping are equally probable, with a constant number of cuts, 10. Consequently, the expected number of cuts using the grouping method in this case is 10, which is also the optimal solution and the true Max‐cut.

**Table 1:** Solutions and the corresponding angle ranges, probabilities and cuts of Figure 4a.

Solution	Angle range	Probability	Cuts
{1, 6, 3, 8} {5, 2, 7, 4}	45°‐	25%	10 (Max‐cut)
{6, 3, 8, 5} {2, 7, 4, 1}	45°	25%	10 (Max‐cut)
{3, 8, 5, 2} {7, 4, 1, 6}	45°	25%	10 (Max‐cut)
{8, 5, 2, 7} {4, 1, 6, 3}	45°	25%	10 (Max‐cut)

Figure 4b shows another 8‐vertex graph with 14 edges. Similarly, from the simulation results (Figure 4f, left), the phase configuration (Figure 4f, right) can be obtained. According to **Table** **2**, the calculated expectation size of cut is 11.106 achieving 92.5% Max‐cut. Moreover, the optimal solution is obtained, reaching 12 cuts.

**Table 2:** Solutions and the corresponding angle ranges, probabilities and cuts of Figure 4b.

Solution	Angle range	Probability	Cuts
{5, 1, 2, 8} {4, 6, 7, 3}	56.9°	31.6%	12 (Max‐cut)
{1, 2, 8, 4} {6, 7, 3, 5}	28.45°	15.8%	11
{2, 8, 4} {6, 7, 3, 5, 1}	18.9°	10.5%	10
{2, 8, 4, 6, 7} {3, 5, 1}	18.9°	10.5%	10
{8, 4, 6, 7} {3, 5, 1, 2}	28.45°	15.8%	11
{4, 6, 7} {3, 5, 1, 2, 8}	14.2°	7.9%	11
{6, 7, 3} {5, 1, 2, 8, 4}	14.2°	7.9%	11

By varying the conductance of resistors in the oscillator network‐based Ising machine, weighted Max‐cut problems can be mapped to the same oscillator‐based Ising machine. Figure 4c,d illustrates two weighted Max‐cut problems with identical patterns but different edge weights, with their experimental results displayed in Figure 4g,h. **Tables** **3** and **4** presents the solution details, including groupings, corresponding probabilities, and number of cuts. The calculated cut number expectations for these problems are 9.174 and 8.656, that is 91.7% and 96.1% of the corresponding Max‐cuts, respectively. Additionally, the grouping with optimal solutions were of relatively large probability in both cases, demonstrating the guaranteed performance in a polynomial time.

**Table 3:** Solutions and the corresponding angle ranges, probabilities and cuts of Figure 4c.

Solution	Angle range	Probability	Cuts
{2, 4} {1, 6, 5, 3}	90°	50%	10 (Max‐cut)
{2, 4, 1, 6} {5, 3}	7.7°	4.3%	7.5
{2, 4, 1} {6, 5, 3}	51°	28.3%	9
{1, 6, 5} {3, 2, 4}	31.3°	17.4%	7.5

**Table 4:** Solutions and the corresponding angle ranges, probabilities and cuts of Figure 4d.

Solution	Angle range	Probability	Cuts
{4, 3, 2} {5, 1, 6}	144°	80%	9 (Max‐cut)
{4, 3} {2, 5, 1, 6}	20.2°	11.2%	7.5
{6, 4} {3, 2, 5, 1}	7.9°	4.4%	6.5
{1, 6, 4} {3, 2, 5}	7.9°	4.4%	7.5

**Table** **5** compares the performance of various oscillator‐network‐based Ising machines. All these machines exhibit relatively fast solving speeds. The oscillator used in this work has a higher frequency compared to others, due to the high resonant frequency MEMS resonator. Consequently, the solving time is significantly reduced. As for the power consumption, a single oscillator consumes approximately 30 mW, with a mere 0.16 nW power for coupling. Regarding the power consumption of individual oscillators, it is relatively large due to the high quiescent current in the triode configuration during the establishment of the oscillator. Nonetheless, the novel grouping technique introduced in this study substantially reduces energy consumption. This strategy eliminates the necessity for additional injection locking signals, consequently reducing the overall energy consumption. Moreover, the versatility of this grouping method allows for its adaptation to an extensive range of oscillator types, rendering it applicable across diverse applications.

**Table 5:** Performance of different types of oscillator‐network‐based Ising machines.

Type of oscillator	Oscillation frequency	Number of nodes	Coupling method	Solution time	Power consumption	Reference
LC	1 MHz	240	R	1 ms	5 W	[11]
LC	50 kHz	4	R	100 µs	/	[28]
Ring	118 MHz	150	Inverter	200 ns^a^ ^)^	23 mW	[51]
VO_2_ phase‐transition	500 MHz	8	C	30 µs	2.56 mW	[24]
Spin Hall	/	100	C	6.8 µs	11.5 mW	[31]
MEMS	9.1 GHz	8	R	90 ns^b^ ^)^	240 mW	this work

^a)^ (Post‐layout simulation of 150 spins with phase contention);

^b)^ This is the synchronization time without consideration of post‐processing steps.

**Figure 5:**
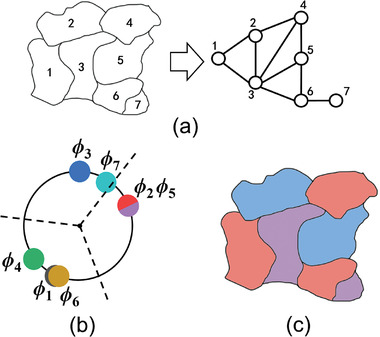
a) Illustration of the 7‐region map and the corresponding oscillator network. b) The phase configuration of (a). c) The successful coloring result.

The graph coloring problem using three colors has also been addressed, with a grouping interval set to 2π/3 (refer to the Supporting Information for a detailed derivation). The graph coloring problem involves assigning colors to a graph's vertices in such a way that no two adjacent vertices share the same color. This well‐known NP‐hard problem is exemplified in this study by considering a map divided into seven regions, which can be represented as a graph with seven vertices (**Figure** **5**a). For regions sharing a border, this is equivalent to having an edge between the corresponding vertices. Consequently, this map can be converted into an oscillator network with ten interconnections.

Once the oscillators’ states stabilize, the system energy reaches its global minimum. The phases of all oscillators lie on a phase circle and this phase circle can be divided into three equal parts with an angle of 120° each, as shown in Figure 5b, representing one of coloring schemes (refer to **Table** **6**). Here we introduce “Points” to count the number of edges that link nodes of different colors, like the number of cuts in the Max‐cut problem. The expected “Points” is 9.379, and the overall probability of successful coloring is 65.5%. In this case, different regions of the map are colored using three colors, as illustrated in Figure 5c. This example demonstrates a successful coloring scheme in which all neighboring sections have different colors.

**Table 6:** Solutions and the corresponding angle ranges, probabilities and points of Figure 5a.

Solution	Angle range	Probability	Points	Success/failure
{3} {7, 2, 5} {1, 6, 4}	55.8°	31%	10	Success
{3, 7} {2, 5} {1, 6, 4}	62.1°	34.5%	10	Success
{3, 7, 2, 5} {1, 6, 4}	12.4°	6.9%	8	Failure
{3, 7, 2, 5} {1, 6} {4}	37.3°	20.7%	8	Failure
{4, 3} {7, 2, 5} {1, 6}	12.4°	6.9%	9	Failure

## 3 Conclusion

In this article, we utilize MEMS oscillators to construct an Ising machine with a high oscillating frequency, resulting in improved solving speed. Furthermore, we introduce an innovative grouping method for a SHIL‐free oscillator‐based Ising machine to tackle combinatorial problems, achieving suboptimal values in polynomial time. This device‐algorithm codesign is applied to both Max‐cut and graph coloring problems, demonstrating a performance guarantee of 0.878 and 0.658 respectively. Due to the fact that the approximate tasks derived from NP problems can be solved in polynomial time using traditional algorithms, our design also provides a remarkable exponential acceleration. Our approach is particularly valuable in scenarios where near‐optimal solutions are acceptable, and low computation latency or time‐to‐solution is essential. By eliminating the binarization step used in SHIL, our method offers benefits for CO problems on the edge.

## 4 Experimental Section

### Devices Fabrication

In this study, the A3 mode MEMS resonators were fabricated using a LiNbO_3_ on insulator (LNOI) wafer comprising a 2 µm SiO_2_ and 600 nm ZX‐LiNbO_3_ layer. The fabrication process began with defining 10/150 nm Cr/Au top electrodes through an evaporation and lift‐off process. Next, a layer of plasma‐enhanced chemical vapor deposition (PECVD) SiO_2_ was deposited and patterned to serve as a hard mask, followed by etching LiNbO_3_ release holes using a Cl_2_‐based inductive coupled plasma (ICP). Subsequently, a 6:1 BOE‐based wet etching process was employed to remove SiO_2_ both above and below the LiNbO_3_ plate, with the etching time carefully controlled to fully suspend the resonators. Last, the liquid beneath the plate was eliminated via a critical point drying (CPD) process.

### Characterization

The fabricated device was then characterized using the Keysight network analyzer (PNA‐X N5247B), A standard 2‐port calibration procedure was also carried out before the device measurement. Then, the measured S‐parameters were imported into ADS for MBVD model fitting and equivalent electrical parameters extraction. Due to our exclusion of the de‐embedding step during the resonator measurement process, this aspect of parasitic effects has been incorporated into the test outcomes. The fitted MBVD model also encompasses this entire dimension.

### Oscillators‐Network Based Ising Machine Implementation

Each individual MEMS serves as a spin node to establish oscillator networks. The couplings between oscillators are realized by resistors standardized at ‐1 kΩ representing one unit weight. Simulations of oscillator‐networks were performed in PSpice under transient mode. An initial voltage (1.5 V) was applied at oscillators to promote the start of oscillation. Then, voltage probes were used to measure the voltage waveforms of all the oscillators after their oscillation states were stable, and finally the corresponding phases were determined.

## Conflict of Interest

The authors declare no conflict of interest.

## Author Contributions

Y.D. and Y.Z. contributed equally to this work. Z.W. and Y.Z. conceived the work. Y.Z., Y.D., X.Z., Y.J., and X.C. contributed to the experiments, simulations and data analysis. Y.D., Y.Z., and Z.W. wrote the manuscript. Y.Y., X.T., Y.C., W.L., and C.S. contributed to the revision of the paper. All authors discussed the results and implications and commented on the manuscript at all stages.

## Supporting information

Supporting Information

## Data Availability

The data that support the findings of this study are available from the corresponding author upon reasonable request.
