# Supplementary material for: MEMS Oscillators‐Network‐Based Ising Machine with Grouping Method
Source: Adv Sci (Weinh). 2024 May 2;11(26):2310096. doi: 10.1002/advs.202310096 (PMC11234442; doi:10.1002/advs.202310096)
Supplement: Supplementary file 1 — Supporting Information [file ADVS-11-2310096-s001.pdf]

ADVANCED SCIENCE
Open Access

Supporting Information

for *Adv. Sci.*, DOI 10.1002/advs.202310096

MEMS Oscillators-Network-Based Ising Machine with Grouping Method

Yi Deng, Yi Zhang, Xinyuan Zhang, Yang Jiang, Xi Chen, Yansong Yang, Xin Tong, Yao Cai, Wenjuan Liu, Chengliang Sun, Dashan Shang, Qing Wang*, Hongyu Yu* and Zhongrui Wang**

Supporting Information

MEMS Oscillators-Network-Based Ising Machine with Grouping Method

Yi Deng†, Yi Zhang†, Xinyuan Zhang, Yang Jiang, Xi Chen, Yansong Yang, Xin Tong, Yao Cai, Wenjuan Liu, Chengliang Sun, Dashan Shang, Qing Wang*, Hongyu Yu*, Zhongrui Wang**

† These authors contributed equally.

Y. Deng, Y. Zhang, X. Zhang, Y. Jiang, X. Chen, Prof. Z. Wang
Department of Electrical and Electronic Engineering, The University of Hong Kong, Pokfulam Road, Hong Kong
E-mail: zrwang@eee.hku.hk; zhangyi97@connect.hku.hk

Y. Deng, Y. Zhang, X. Zhang, Y. Jiang, X. Chen, Prof. Z. Wang
ACCESS – AI Chip Center for Emerging Smart Systems, InnoHK Centers, Hong Kong Science Park, Hong Kong

Y. Zhang, Y. Jiang, Prof. Q. Wang, Prof. H. Yu
School of Microelectronics, Southern University of Science and Technology, Shenzhen, 518055, China
Email: wangq7@sustech.edu.cn; yuhy@sustech.edu.cn

Prof. Y. Yang
Department of Electronic and Computing Engineering, Hong Kong University of Science and Technology, Hong Kong

X. Tong, Y. Cai, W. Liu, C. Sun
Institute of Technological Sciences, Wuhan University, Wuhan 430072, China

Prof. D. Shang
Institute of Microelectronics, Chinese Academy of Sciences, Beijing, 100029, China
Email: shangdashan@ime.ac.cn

Keywords: MEMS Oscillator; Ising Machine; Semidefinite Programming Relaxation; Combinatorial Optimization; Max-Cut

Supporting Note 1. Comparison of the state-of-the-art (SoA) MEMS oscillators

Table S1 summarized the SoA MEMS oscillators operating above 5 GHz. Owing to the high *Q* value and figure-of-merit (FOM) nature of the $LiNbO_3$ resonator, the proposed design might exhibit a comparatively low phase noise after careful design and optimization. Significant advancements in the field of MEMS oscillators, particularly those employing resonant-fin-transistor (RFT) architectures, have led to remarkable achievements. With reported operational frequencies surpassing the 30 GHz benchmark, these developments highlight the burgeoning potential for improving solution speeds in this domain. This progress in academic research underscores the importance of continued exploration and optimization within the context of MEMS oscillator technology.

Table S1. Comparison of the SoA MEMS oscillators above 5 GHz

		[1]	[2]	[3] simulation	[4] simulation	[5] simulation	[6]	[7]	[8] simulation
Resonator		$LiNbO_3$ A3 mode	$LiNbO_3$ A3 mode	RFT	RFT	RFT	FBAR 3rd tone	FBAR fundamental	FBAR 3rd tone
IC Process		Discrete	65 nm CMOS	14 nm CMOS	14 nm CMOS	16 nm CMOS	Discrete	0.35 μm BiCMOS	90 nm CMOS
Oscillation Frequency (GHz)		12.9	8.6	30	30	33.3	7	5.46	6.3
Footprint (mm^2)		0.01 (resonator)	0.016 (resonator)	0.0025	/	/	/	0.034 (resonator)	/
DC Power (mW)		20	10.2	2	5.5	1.24	16.2	12.7	0.475
Phase Noise (dBc/Hz)	100 kHz	-111	-113	/	/	/	-80	-117.7	/
	1 MHz	-131	-147	-132	-144	-82	-110	/	-110

FOM (dBc/Hz)	100 kHz	200.2	201.6	217	/	170	164.8	201.4	/

Supporting Note 2. Max-cut problem

The Max-cut problem can be matched with the Ising Hamiltonian equation directly. Firstly, we separate the vertices into 2 groups (V_1 and V_2), and set J_{ij} as the opposite value of w_{ij}, namely $J_{ij} = -w_{ij}$, where J_{ij} is the term in Ising Hamiltonian equation and w_{ij} is the weight of edges between vertex *i* and vertex *j*. Therefore, the value of cut S_{cut} is defined as follows:

$$S_{cut} = \sum_{i \in V_1,\ j \in V_2} w_{ij} \quad . (1)$$

Then assigning binary spin values, $s_i = +1$ if $s_i \in V_1$ and $s_i = -1$ if $s_i \in V_2$. The S_{cut} can be rewritten as follows:

$$S_{cut} = \sum_{i<j} w_{ij} \frac{1 - s_i s_j}{2} \quad . (2)$$

Combining (1) and (2), the S_{cut} can be calculated as follows:

$$\begin{aligned} S_{cut} &= \frac{1}{2}\sum_{i<j} w_{ij}(1 - s_i s_j) \\ &= \frac{1}{2}\sum_{i<j} w_{ij} - \frac{1}{2}\sum_{i<j} w_{ij} s_i s_j \end{aligned} \quad (3)$$

where $\sum_{i<j} w_{ij}$ is the sum of all weights which is a constant, so S_{cut} will be maximized when the Ising Hamiltonian *H* is minimized.

Supporting Note 3. Size of cut under grouping method

The Max-cut problem can be matched with the Ising Hamilto

In the following part, the size of cut under grouping method instead of SHIL will be obtained through derivation. In addition, the relation between the size of cut obtained by grouping method and the true Max-cut will be gained through the transitivity of inequalities.

Without SHIL, the oscillator phases will be continuous. It can be assumed that each oscillator's state is a unit vector whose angle corresponds to its phase. Then, assuming $X_{ij} = \vec{s}_i \vec{s}_j = \cos\Delta\phi_{ij}$, the continuous S_{cut} can be expressed as follows:

$$(S_{cut})_{continous} = \frac{1}{2}\sum_{i<j} w_{ij}(1 - X_{ij}) \quad . (4)$$

When adding SHIL, the $s_i s_j = \pm 1$, so the S_{cut} under SHIL is written as follows:

$$(S_{cut})_{true} = \frac{1}{2}\sum_{i<j} w_{ij}(1 - X_{ij}),\ X_{ij} = \pm 1 \quad . (5)$$

And equation (5) is the true S_{cut} obtained from the Ising machine with SHIL. Because the oscillator system energy can achieve a lower energy without the constraint of SHIL, we have

$$(S_{cut})_{continous} \geqslant (S_{cut})_{true} \quad . (6)$$

As for the oscillators with various phases, we can divide them into two groups randomly using a straight line passing through the center of the circle. The probability of two oscillators being in different groups is $\frac{\Delta\phi_{ij}}{\pi}$, while the probability of two oscillators being in the same group is $1 - \frac{\Delta\phi_{ij}}{\pi}$. The value of $s_i s_j$ is -1 and +1, respectively, when the two oscillators are in different groups and in the same group. Therefore, the expectation value of Ising Hamiltonian under such a grouping method can be expressed as follows:

$$\begin{aligned} E[H] &= \sum_{i<j} w_{ij}\left(-\frac{\Delta\phi_{ij}}{\pi} + 1 - \frac{\Delta\phi_{ij}}{\pi}\right) \\ &= \sum_{i<j} w_{ij}\left(1 - \frac{2\Delta\phi_{ij}}{\pi}\right) \\ &= \sum_{i<j} w_{ij}\left(1 - \frac{2}{\pi}\arccos X_{ij}\right) \\ &= \frac{2}{\pi}\sum_{i<j} w_{ij}\arcsin X_{ij} \end{aligned} \quad . (7)$$

From equation (7), the S_{cut} under this grouping method can be written as

$$(S_{cut})_{grouping} = \frac{1}{2}\sum_{i<j} w_{ij} - \frac{1}{2}E[H]$$
$$= \frac{1}{2}\sum_{i<j} w_{ij}\frac{2}{\pi}\arccos X_{ij} \quad . (8)$$

Since the S_{cut} from grouping method cannot exceed the true S_{cut}, combining (6) we can get

$$(S_{cut})_{grouping} \leqslant (S_{cut})_{ture} \leqslant (S_{cut})_{continous} \quad . (9)$$

Assuming there is a α making the inequality as follow:

$$\frac{2}{\pi}\arccos X_{ij} \geqslant \alpha(1 - X_{ij}), \quad X_{ij} \in [-1, 1] \quad , (10)$$

except for the case $X_{ij} = 1$, we have

$$\alpha \leqslant \frac{2}{\pi}\frac{\arccos X_{ij}}{1 - X_{ij}}, \quad X_{ij} \in [-1, 1) \quad . (11)$$

In the right side of the inequality (11), there is a minimum value approximately equaling to 0.878 in the domain of definition. As a result, in addition to the case $X_{ij} = 1$, we can get

$$\frac{2}{\pi}\arccos X_{ij} \geqslant 0.878(1 - X_{ij}), \; X_{ij} \in [-1, 1] \quad . (12)$$

According to (12), in the case all the weights are positive, namely $w_{ij} > 0$, we can get the relation associated with (4) and (8):

$$0.878(S_{cut})_{continous} \leqslant (S_{cut})_{grouping} \quad . (13)$$

Combining inequalities (9) and (13), finally we can get

$$0.878(S_{cut})_{ture} \leqslant (S_{cut})_{grouping} \quad . (14)$$

which demonstrates that the SHIL-free Ising machine utilizing a grouping method can achieve a minimum expectation value of 0.878 for the true Max-cut on solving positive-weight Max-cut problems.

Supporting Note 4. Graph coloring problem

The graph coloring problem requires each pair of boarding graph sections to be colored with different colors. The graph to be colored can be transformed as an undirected graph with some vertices and edges. Some graph coloring problems can be solved using 3 colors. Firstly, we regard each vertex s_i as a unit vector which can be only 3 discrete angles (0, 2π/3 and 4π/3). The vertices are grouped into 3 groups, V_1, V_2 and V_3 according to their phases. And the edges E across the same groups belongs to V_{11}, V_{22} and V_{33}, while the edges E across different groups belongs to V_{12}, V_{13} and V_{23}. If we see the problem as a Max-cut problem in which an edge across different groups is a cut, we have $S_{cut} = \sum_{E \in V_{12}} w_{ij} + \sum_{E \in V_{13}} w_{ij} + \sum_{E \in V_{23}} w_{ij}$. Similarly, we assume that there is an Ising Hamiltonian H and set $J_{ij} = - w_{ij}$, where J_{ij} is the term in Ising Hamiltonian equation and w_{ij} is the weight of edges between two vertices. We have

$$
\begin{aligned}
H &= -\sum_{i<j} J_{ij} \vec{s}_i \vec{s}_j \\
&= \sum_{i<j} w_{ij} \vec{s}_i \vec{s}_j \\
&= \sum_{E \in V_{11}} w_{ij} + \sum_{E \in V_{22}} w_{ij} + \sum_{E \in V_{33}} w_{ij} - \frac{1}{2}\sum_{E \in V_{12}} w_{ij} - \frac{1}{2}\sum_{E \in V_{13}} w_{ij} - \frac{1}{2}\sum_{E \in V_{23}} w_{ij} \\
&= \sum_{i<j} w_{ij} - \frac{3}{2}\left(\sum_{E \in V_{12}} w_{ij} + \sum_{E \in V_{13}} w_{ij} + \sum_{E \in V_{23}} w_{ij}\right) \\
&= \sum_{i<j} w_{ij} - \frac{3}{2} S_{cut}
\end{aligned}
\quad . (15)
$$

So, the S_{cut} can be rewritten as follows:

$$
S_{cut} = \frac{2}{3}\sum_{i<j} w_{ij} - \frac{2}{3}\sum_{i<j} w_{ij} \vec{s}_i \vec{s}_j \quad . (16)
$$

From (15) and (16), we know S_{cut} will be maximized when the Ising Hamiltonian H is minimized. And the largest S_{cut} is $\sum_{i<j} w_{ij}$ when all the neighboring vertices are in different groups. That is, the neighboring group sections are assigned to different colors from the view of graph coloring problems.

Supporting Note 5. Graph coloring problem under grouping method

When using oscillator network to solve graph coloring problems, it can be assumed that each oscillator's state is a unit vector whose angle corresponds to its phase. Then, assuming $X_{ij} = \vec{s}_i \vec{s}_j = \cos\Delta\phi_{ij}$, there is a continuous S_{cut} that can be expressed as follows:

$$(S_{cut})_{continous} = \frac{2}{3}\sum_{i<j} w_{ij}(1 - X_{ij}) \quad . (17)$$

When forcing the phases being 3 discrete values like 0, 2π/3 and 4π/3, the $\vec{s}_i \vec{s}_j = 1 \text{ or } -\frac{1}{2}$, so the true S_{cut} is written as follows:

$$(S_{cut})_{true} = \frac{2}{3}\sum_{i<j} w_{ij}(1 - X_{ij}),\ X_{ij} = 1 \text{ or } -\frac{1}{2} \quad . (18)$$

The true S_{cut} in (18) is the same as (16). Because the oscillator system energy can achieve a lower energy without constraint, similarly we have:

$$(S_{cut})_{continous} \geqslant (S_{cut})_{true} \quad . (19)$$

As for the oscillators with continuous phases, we can divide them into three groups randomly by a range of 2π/3. When $\Delta\phi_{ij} \leqslant \frac{2\pi}{3}$, the probability of two oscillators being in different groups is $\frac{3\Delta\phi_{ij}}{2\pi}$, while the probability of two oscillators being in the same group is $1 - \frac{3\Delta\phi_{ij}}{2\pi}$. When $\Delta\phi_{ij} > \frac{2\pi}{3}$, the probability of two oscillators being in different groups is 1, while the probability of two oscillators being in the same group is 0. The value of $\vec{s}_i \vec{s}_j$ is -1/2 and +1, respectively, when the two oscillators are in different groups and in the same group. Therefore, the expectation value of Ising Hamiltonian under such a grouping method can be expressed as follows:

$$\begin{aligned} E[H] &= \sum_{i<j} w_{ij}\left[-\frac{1}{2} \times \frac{3\Delta\phi_{ij}}{2\pi} + 1 \times \left(1 - \frac{3\Delta\phi_{ij}}{2\pi}\right)\right] \\ &= \sum_{i<j} w_{ij}\left(1 - \frac{9\Delta\phi_{ij}}{4\pi}\right) \\ &= \sum_{i<j} w_{ij}\left(1 - \frac{9}{4\pi}\arccos X_{ij}\right) \end{aligned} \quad . (20)$$

From (20), the S_{cut} under this grouping method can be written as

$$(S_{cut})_{grouping} = \frac{2}{3}\sum_{i<j} w_{ij} - \frac{2}{3}E[H]$$
$$= \frac{2}{3}\sum_{i<j} w_{ij}\frac{9}{4\pi}\arccos X_{ij} \quad . (21)$$

Since the S_{cut} from grouping method cannot exceed the true S_{cut}, combining (19) we can get

$$(S_{cut})_{grouping} \leqslant (S_{cut})_{ture} \leqslant (S_{cut})_{continous} \quad . (22)$$

Also, there is a β making the inequality as follow:

$$\frac{9}{4\pi}\arccos X_{ij} \geqslant \beta(1 - X_{ij}),\ X_{ij} \in [-1,\ 1] \quad . (23)$$

Just like (11) and (12), we have

$$\frac{9}{4\pi}\arccos X_{ij} \geqslant 0.987(1 - X_{ij}),\ X_{ij} \in [-1, 1] \quad . (24)$$

According to (24), we can get the relation associated with (17) and (21): $0.987(S_{cut})_{continous} \leqslant (S_{cut})_{grouping}$. Combined with (22), we can get:

$$0.987(S_{cut})_{true} \leqslant (S_{cut})_{grouping} \quad . (25)$$

However, when $\Delta\phi_{ij} > \frac{2\pi}{3}$, the S_{cut} under grouping in (21) will reach $\frac{3}{2}\sum_{i<j} w_{ij}$ in the worst case, which is 3/2 times the true S_{cut}, $\sum_{i<j} w_{ij}$. As a result, the (25) should be modified when considering the worst case:

$$0.658(S_{cut})_{true} \leqslant (S_{cut})_{grouping} \quad . (26)$$

which means the grouping method has a performance guarantee of 0.658 when considering the number of successful colored edges on 3-color graph coloring problems.

Supporting Note 6. Probabilities of solutions under grouping method

For the grouping method, we first record the phase positions of oscillators using an oscilloscope, on the phase circle. The phase circle is then divided using a straight line passing through the center, with the effective angle range of this line spanning from 0° to 180°. Each solution is

associated with a specific angle range, which can be translated to its corresponding probability. This part will present the calculation process of angle range and probability of one solution.

In the case of n oscillators, if we assign each oscillator a number, there must be a sequence of loop for n oscillators on a unit circle. For example, when n = 5, the sequence of loop for oscillators can be {…, 1, 2, 4, 5, 3, 1, 2, 4, 5, 3, 1, 2, 4, …}, and the corresponding sequence of loop for phases is {…, ϕ_1, ϕ_2, ϕ_4, ϕ_5, ϕ_3, ϕ_1, ϕ_2, ϕ_4, ϕ_5, ϕ_3, ϕ_1, ϕ_2, ϕ_4, …}. As to any solution $\{a_m\} = \{a_1, a_2, a_3, \ldots, a_m\}$ and $\{b_k\} = \{b_1, b_2, b_3, \ldots, b_k\}$, where $m + k = n$ and $\{a_m\}$ and $\{b_k\}$ are part of the sequence of loop for oscillators, the sequence of number $\{c_n\} = \{a_1, a_2, a_3, \ldots, a_m, b_1, b_2, b_3, \ldots, b_k\}$ must follow the sequence of loop for oscillators, such as $\{a_m\}$ = {1, 2, 4}, $\{b_k\}$ = {5, 3} and $\{c_n\}$ = {1, 2, 4, 5, 3} when n = 5. Then we can assume that $0 \le \phi_{a1} \le \phi_{a2} \le \phi_{a3} \le \ldots \le \phi_{am} \le \phi_{b1} \le \phi_{b2} \le \phi_{b3} \le \ldots \le \phi_{bk} \le 2\pi$, based on which we can obtain the probability P of this solution.

If $\phi_{am} - \phi_{a1} \ge \pi$ or $\phi_{bk} - \phi_{b1} \ge \pi$, then $P = 0$. Because oscillator a_m and a_1 must not be in the same group or b_k and b_1 must not be in the same group. The following cases will be discussed under the condition $\phi_{am} - \phi_{a1} < \pi$ and $\phi_{bk} - \phi_{b1} < \pi$. There is a $\{\Delta\phi_{a1bk}, \Delta\phi_{amb1}\}_{\min}$. Assuming $\{\Delta\phi_{a1bk}, \Delta\phi_{amb1}\}_{\min} = \Delta\phi_{ab}$, if $\Delta\phi_{ab} + \Delta\phi_{a1am} \le \pi$ and $\Delta\phi_{ab} + \Delta\phi_{b1bk} \le \pi$, $P = \Delta\phi_{ab}/\pi$; if $\Delta\phi_{ab} + \Delta\phi_{a1am} > \pi$ and $\Delta\phi_{ab} + \Delta\phi_{b1bk} \le \pi$, $P = (\pi - \Delta\phi_{a1am})/\pi$; if $\Delta\phi_{ab} + \Delta\phi_{a1am} \le \pi$ and $\Delta\phi_{ab} + \Delta\phi_{b1bk} > \pi$, $P = (\pi - \Delta\phi_{b1bk})/\pi$.

Figure S1 illustrates an example of grouping six phases into two groups. For grouping into {2, 4} {1, 6, 5, 3}, the angle range is 90°, the corresponding probability is 50%. For grouping into {2, 4, 1} {6, 5, 3}, the angle range is 51°, the corresponding probability is 28.3%.

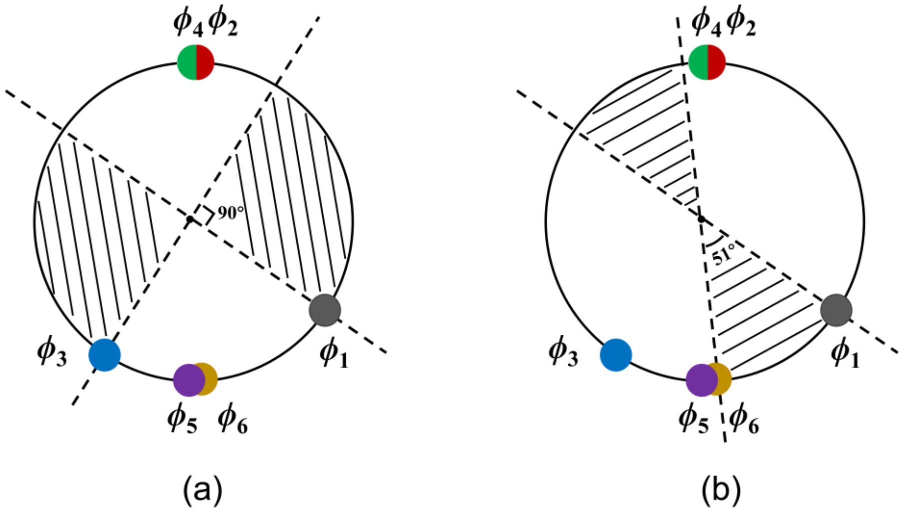

Figure S1 (a) For grouping into {2, 4} {1, 6, 5, 3}, the angle range is 90°, the corresponding probability is 50% (b) For grouping into {2, 4, 1} {6, 5, 3}, the angle range is 51°, the corresponding probability is 28.3%.

References

[1] A. Kourani, Y. Yang, S. Gong, *IEEE Microw. Wirel. Co.* **2020**, *30*, 681.

[2] A. Kourani, Y. Yang, S. Gong, *IEEE Trans. Ultrason. Ferr.* **2021**, *68*, 1994.

[3] A. Srivastava, B. Chatterjee, U. Rawat, Y. He, D. Weinstein, S. Sen, *IEEE Trans. Circuits Syst. II Express Briefs.* **2021**, *68*, 1108.

[4] A. Srivastava, B. Chatterjee, D. Weinstein, S. Sen, in *IEEE 35th Int. Symp. VLSI Des. & 21st Int. Conf. Embed. Syst. (VLSID)*, **2022,** pp. 192-197.

[5] R. Hudeczek, E. Hager, P. Baumgartner, H. Pretl, *IEEE Access* **2022**, *13*, 64388.

[6] M. ElBarkouky, G. Vandersteen, P. Wambacq, Y. Rolain, in *IEEE European Microwave Conference (EuMC)*, **2009**, pp. 318-321.

[7] M. Aissi, E. Tournier, M. A. Dubois, G. Parat, R. Plana, in *IEEE International Solid State Circuits Conference-Digest of Technical Papers*, **2006**, pp. 1228-1235.

[8] M. ElBarkouky, P. Wambacq, Y. Rolain, in *IEEE Ph. D Research in Microelectronics and Electronics Conference*, **2007**, pp. 61-64.
